# Survival of the Fittest: Overcoming Oxidative Stress at the Extremes of Acid, Heat and Metal

**DOI:** 10.3390/life2030229

**Published:** 2012-08-23

**Authors:** Yukari Maezato, Paul Blum

**Affiliations:** Beadle Center for Genetics, University of Nebraska, Lincoln, NE 68588-0666, USA; E-Mail: yukarimaezato@hotmail.com

**Keywords:** archaea, ecology, oxidative stress, metals, natural habitats

## Abstract

The habitat of metal respiring acidothermophilic lithoautotrophs is perhaps the most oxidizing environment yet identified. Geothermal heat, sulfuric acid and transition metals contribute both individually and synergistically under aerobic conditions to create this niche. Sulfuric acid and metals originating from sulfidic ores catalyze oxidative reactions attacking microbial cell surfaces including lipids, proteins and glycosyl groups. Sulfuric acid also promotes hydrocarbon dehydration contributing to the formation of black “burnt” carbon. Oxidative reactions leading to abstraction of electrons is further impacted by heat through an increase in the proportion of reactant molecules with sufficient energy to react. Collectively these factors and particularly those related to metals must be overcome by thermoacidophilic lithoautotrophs in order for them to survive and proliferate. The necessary mechanisms to achieve this goal are largely unknown however mechanistics insights have been gained through genomic studies. This review focuses on the specific role of metals in this extreme environment with an emphasis on resistance mechanisms in Archaea.

## 1. Introduction

The presence of heavy metals in extreme microbial habitats is common. This juxtaposition offers an important opportunity to investigate resistance and toxicity of diverse heavy metals towards natural communities and individual taxa. Heavy metal resistance in bacteria has been widely reviewed [1,2,3,4]. For bacteria, the ecology, chemistry and biologic mechanisms of resistance associated with arsenic, selenium and copper have also been described [3,5]. Heavy metals promote highly oxidative environments which lead to a differential level of toxicity of the metal [6,7]. The response to toxic elements involves diverse strategies including: redox-based metal transformation [8]; trafficking within the cytoplasm mediated by metal chaperones [9,10]; protein sequestration [11,12]; metal efflux [8]; and by metal-phosphate symport [13,14]. Horizontal gene transfer also plays a crucial part in the evolution of heavy metal resistance within bacterial communities [15]. Archaea also occupy diverse extreme habitats including those that are metal rich. Examples include acidic and sulfidic geothermal pools and soils, marine hydrothermal vents emanating from metal rich ores, and hypersaline pools and soil crusts saturated with metals and other elements. In some of these habitats particularly ore heaps, thermophilic archaea proliferate because of the intrinsic heat generated by chemical and biological oxidation that also promotes solubilization of metal sulfide complexes. However, though archaea flourish in such environments, much less is understood about their mechanisms of survival. Analysis of extant archaeal genomes identifies many genes involved with defenses againts toxic metals while studies using model archaeal taxa have begun to yield mechanistic details underlying metal resistance. Therefore, this review examines the literature related to metal biology of archaea. 

## 2. Ecological Considerations of Archaea in Metal Rich Environments

Many toxic metals including copper (Cu) cadmium (Cd), zinc (Zn), uranium (U) and arsenic (As) are commonly called heavy metals but are also assigned to the category of “soft metals” because of their physical properties and their large ratio of ionic charge to ionic radius [16]. In contrast, alkali metals and alkali earth metals such as potassium (K) and calcium (Ca) are placed in the category of “hard metals”. The stronger interaction of soft metals with proteins compared to the weaker ionic interaction with hard metals, is one factor underlying their toxicity [16] and is a reflection of the tendency of soft acids and bases to interact [17]. Despite this effect, soft metals are also important at low concentrations in diverse biological processes [18] such as those required between enzymes and their co-factors. To balance the critical need for trace metals against their potential toxicity, archaea have evolved multiple regulatory mechanisms to control metal exposure in their environments.

Archaea, representing one of the three domains of life, have been found in diverse environments [19]. The discovery and isolation of numerous archaeal species from environments with high concentrations of heavy metals which also contributes to the highly oxidative environment such as mining sites, salterns, and metal contaminated soils, has accelerated interest in studying metal resistance in these organisms. Importantly, archaea as a fraction of total cells are abundant in many habitats from benign to extreme (Table 1). The apparent abundance of archaeal cells implicates them as agents capable of mediating metal transformations. The best characterized archaeal biotypes provide an important context for much of the information that is available linking heavy metals with these organisms. While these biotypes are generalizations that have been expanded through culture-independent studies on archaeal biodiversity, they continue as physiologic paradigms providing a basis for mechanistic information. 

**Table 1:** Relative abundance of archaea in the environment.

Habitat	Abundance(Maximum)	Dominant archaeal type	Method of determination	Citation
**Aquatic **				
Marine	24%–34% of total prokaryotic rRNA	Crenarchaeota	Measuring of amplification of group specific ribosomal RNA	[20]
Shallow water hydrothermal vent	12% of total prokaryotic rRNA sampled	Euryarchaeota (*i.e.*, Thermococcus, Pyrococcus,Pyrobaculum, methanococcus)	Ribosomal RNA hybridization, Fluorescent in situ Hybridization (FISH)	[21]
Deep-sea Sulfide Chimney	33%–60%	Marine group I Crenarchaeota and Euryarchaeota (*i.e.*, Thermococcales)	Fluorescent in situ Hybridization (FISH), 16SrRNA analysis, and RFLP anlaysis	[22]
Holo-oligomictic Lake	47%		Catalyzed Reporter Deposition- Fluorescence In Situ Hybridization (CARD-FISH) analysis	[23]
**Terrestrial**				
Geothermal, solfatara	70%	Crenarchaeota (*i.e.*, Sulfolobales)	Fluorescent in situ Hybridization (FISH) analysis	[24]
Solar (Saltern) pond	80%–86% of total prokaryotic rRNA	Euryarchaeaota (*i.e.*, Haloarchaea)	DAPI counts and Fluorescent in situ Hybridization (FISH) analysis	[25]
Soil	~2% of total sampled 16S rRNA	Thaumarchaeota	16S rRNA analysis and barcoded pyrosequencing analysis	[26,27]
**Metal Rich environment**				
Acid mine drainage (AMD)/Mining sites	Up to 80% of sampled 16S rRNA	Euryarchaea (*i.e.*, Ferroplasma)	16S rRNA library sequencing and Fluorescent in situ Hybridization (FISH) analysis	[28,29,30]

### 2.1. Haloarchaea

Halophilic archaea, including the obligate and extremely halophilic taxa, belong to the phylum *Euryarchaeota*. They occur in areas with high concentrations of salt (>2 M), such as the Dead Sea, salt lakes, inland seas, and evaporating ponds of seawater. These hypersaline habitats are also rich in heavy metals [31,32], and many extreme halophiles have developed strategies to overcome metal toxicity [33]. For example, *Halobacterium *strain NRC-1 has multiple mechanisms for responding to arsenic. In the presence of arsenite (As (III)), these organisms oxidize the metal and then export it via metal specific translocators. Alternatively they alkylate (methylate) this metal as an alternative means of detoxification [32]. High level arsenic resistance in *Halobacterium *strain NRC-1 is mediated by the plasmid encoded *ars* operons (*arsADRC*, and *M*), rather than the chromosome encoded *arsB* gene [32]. 

### 2.2. Methanoarchaea

Methanogenic archaea produce methane and also are classified as members of the *Euryarchaeota*. Methanoarchaea are found in diverse environments such as deep sea sediments [34], polar caps [20], agricultural soils, and sewage sludge [35]. However, the molecular mechanisms of heavy metal resistance in methanogens are not well characterized. Bioinformatic surveys of various genomes reveal the presence of gene homologs involved with heavy metal resistance. These include arsenic resistance genes (*arsA*, *C*, *D*, *R * and *M*) and copper resistance genes (*copA*, *R*). One molecular genetic study of copper resistance in *Methanobacterium bryantii* suggests involvement of the copper inducible protein Crx (copper response extracellular protein) [36]. A cadmium and copper resistant novel species, *Methanocalculus pumilus*, has also been described [37] though the responsible mechanisms are not yet characterized. Methanogens are important facilitators of geochemical cycling of various heavy metals through their ability to form methylated forms of heavy metals in the environment [38]. Methylation of heavy metals is probably accomplished enzymatically through the action of putative methytransferases such as ArsM [39]. 

### 2.3. Hyperthermophilic Archaea

In general, hyperthemophilic archaea and bacteria grow at or above 80 °C. Hyperthermophilic archaea inhabit high temperature habitats such as deep sea hydrothermal vents, geothermal springs and solfataras (sulfur rich volcanic pond) as well as various metal mining sites [40,41,42,43]. Many of the thermophilic and hyperthermophilic archaea are also extremely acidophilic and tolerate pH values less than one [44]. The occurrence of heavy metals (e.g., Cu, Hg, Cd, and As) in hydrothermal and geothermal habitats is well known [45,46,47,48], and the microbes residing in such niches are faced with the constant challenge of coping with toxic metals. For example *Metallosphaera prunae *an extremely thermoacidophilic species belonging to the phylum *Crenarchaeota*, was isolated from a uranium mining site [42]. Uranium (U) occurs primarily in two redox states, U^+4^ is insoluble while U^+6^ is soluble and may have implications for metal resistance mechanisms. The mesophilic acidophile, *Ferroplasma acidarmanus*, exhibits bacterial-like arsenite (As(III)) resistance mechanisms involving *arsA* (ATPase), *arsB* (efflux pump for As (III)), *arsD* (chaperone) and *arsR* (transcriptional regulator). However, resistance to arsenate (As(V)) is likely to involve a novel mechanism because the *F. acidarmanus* genome lacks an apparent arsenate reductase (*arsC*) necessary to overcome high intracellular levels of oxidized metal [49] perhaps an example of a divergent but related activity. 

Other acidophilic archaea notably the hyperthermoacidophile, *Sulfolobus solfataricus*, occur in metal rich habitats such as Coso Hot Springs (CSH) an abandoned mercury mining area at the edge of the Mojave desert in southeastern California, USA. This natural geothermal environment contains high concentrations of Hg ranging from 2.0 mg/li of pool water to nearly 1 g/kg sediment derived from cinnabar (mercuric sulfide) the primary ore of mercury [24]. Mercury is a toxic metal for all three domains of life with minimal inhibitory concentrations (MIC) ranging from micromolar to millimolar concentrations depending on the domain. The mechanism of toxicity of Hg(II)) towards *S. solfataricus* arises from the inactivation of TFIIB one of the core archaeal generalized transcription factors required for gene transcription [50]. This mechanism is identical to that observed in eukaryotes and unlike that observed in bacteria. Unlike all eukaryotes however, *S. solfataricus* also encodes genes that detoxify this metal. A mercury resistance operon including *merR* (regulator), *merH* (TRASH domain, metal chaperone), and *merA* (metal reductase) was characterized using directed recombination to generate gene knockouts [51]. These studies demonstrated that *merA* was required for metal resistance and that the transcription of these genes was responsive to Hg(II) exposure [51,52]. 

## 3. Levels of Resistance Towards Heavy Metals

Archaeal taxa especially acidophiles, exhibit relatively high tolerance to many heavy metals. For example, the highly arsenic resistant *Ferroplasma acidarmanus* can tolerate ~130 mM As in its environment (Table 2). Hyperthermophilic archaea also exhibit higher resistance towards various metals such as Zn, Cu and Cd. Interestingly, the MIC of As in haloarchaea strain NRC1 is relatively high. This may arise from the presence of multiple As resistance mechanisms including the plasmid and chromosome encoded As efflux system and the As methylation detoxification system (ArsM). Although an ArsM like protein is also evident in hyperthermophilic archaeal genomes, ArsC homologs are lacking suggesting there are either divergent orthologs or an alternative resistance mechanism. 

**Table 2:** Minimum inhibitory concentration of metals.

Microorganism	MIC (mM)							Citation
	Zn (II)	Cu (II)	Ag (I)	Hg (II)	As (III)	U (VI)	Cd (II)	
**Haloarchaea**								
*Halobacterium sp.*	0.5	1–2.5	0.5	0.01	~20	NA^a^	≤2.5	[53]
*Halocula sp.*	0.05	2.5	0.05	0.01	10	NA	0.05	[53]
**Hyperthermophilic archaea**								
*Sulfolobus solfataricus*	50	1~5	0.008	0.002	NA	NA	10	[54,55]
*Sulfolobus metallicus*	300	16	0.09	0.05	1.3	0.4	0.9	[56]
*Metallosphaera sedula*	150	100	0.09	0.05	1	0.4	1	[43]
**Methanoarchaea**								
*Methanocalculus pumilus*	NA	1	NA	NA	NA	NA	1	[37]
*Methanobacterium bryantii*	NA	~1.8	NA	NA	NA	NA	NA	[57]
**Acidophilic archaea**								
*Ferroplasma acidarmanus Fer1*	NA	312	NA	NA	133	NA	1	[49,54,58,59]
**Bacteria**								
*Acidithiobacillus ferrooxidans*	750	~800	0.9	0.5	1.3	0.4	0.09	[43,49]
*E. coli *ASU7	10	1~3	~>1	0.013	1	2	5	[7,60,61,62]

Extremely thermoacidophilic members of *Metallosphaera* exhibit among the higher levels of resistance towards copper of all prokaryotes (Table 2). This distinguishing trait is of interest because of its importance to the mining industry (see below). In the case of copper resistance, *copA* (efflux pump) knockout mutants in *M. sedula* show reduced levels of resistance to cupric ion (Cu (II)), however such mutants retain considerable levels of metal resistance [63]. As is the case for As resistance in *Haloarchaea*, perhaps there are multiple mechanisms orchestrating copper resistance in *M. sedula*. 

In contrast to copper resistance in *M. sedula* where the trait correlates with environmental metal abundance, this is not the case with Hg and the related *S. solfataricus*. Since Hg levels are elevated in the Coso Hot Springs habitat, it was likely that *S. solfataricus* could elaborate high levels of mercury resistance. Unexpectedly, endogenous isolates of this organism were not unusually metal resistant (0.3–2 μM) and instead were comparable to other mercury resistant bacterial species [24,64,65]. These findings indicate that other mechanisms perhaps dependent on solute transport or internal redox homeostasis must be operative that spare endogenous archaea from metal toxicity. 

## 4. Strategies of Heavy Metal Resistance of the Archaea

Prolonged ecologic and biotechnologic interests in the archaea promote ongoing studies of metal resistance in these organisms. Development of bioremediation strategies have also motivated detailed studies of archaeal metal resistance and the biodiversity of archaeal taxa resident in heavy metal mining sites and metal contaminated habitats [28,42,43,66,67]. The occurrence of thermophilic taxa in some of these habitats particularly ore heaps arises from the intrinsic heat generated by chemical and biological oxidation. This creates a variable environment which develops into one containing high concentrations of solubilized metals dissociated from metal sulfide complexes. 

Whether in ore heaps or saline ponds, changes in heavy metal concentration elicit differential gene regulation in archaea. Comparisons of global gene expression patterns arising from heavy metal challenges in *Halobacterium sp. *strain NRC-1 have been demonstrated [33]. In some case, identical genes are up-regulated by different metals such as *yvgX* in the presence of Cu and Zn, and *pstC1* in the presence of cobalt (Co) and nickle (Ni). On the other hand, some genes are uniquely up-regulated in the presence of only certain metals such as *potA1* and *hemeV2* in the presence of manganese (Mn) [33]. This suggests that the cellular perception of metal involves complex signal transduction mechanisms. Multiple mechanisms of Cu resistance also have been identified based on gene expression analysis in the hyperthermophilic acidophile *S. solfataricus*. These include a copper efflux system by *copA* and *B* (P-type ATPase), *copR* (regulator), and *copT* (chaperone) [68,69,70], and an inorganic polyphosphate transport system in the related *S. metallicus* [13]. The distribution of copper resistance genes among archaea is broad. Many archaeal genomes encode *copA* (P-type ATPase) homologs. However, while many bacterial taxa have metal resistance genes encoded on both plasmids and the genome, there are no CopA homologs annotated as being encoded on archaeal plasmids with the exception of *Haloarcula marismortui* ATCC 43049 mega plasmid pNG600. In this partcular case, five CopA homologs are apparent on this plasmid.

**Figure 1:**
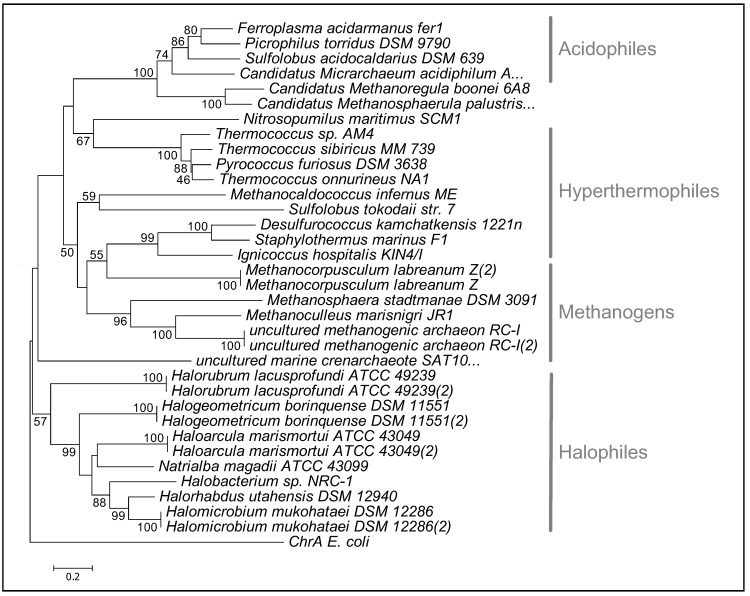
Protein phylogenetic tree of archaeal ChrR. The tree was constructed using MEGA 4.0. The bootstrap values were based on 1000 replicates, and *E. coli* ChrA was used as an outgroup. Bootstrap value greater than 50% are shown.

Hexavalant chromium (Cr(VI)) is another example of a heavy metal with environmental relevance. Like mercury, chromium is not an essential trace metal. Various studies have reported that many taxa have the ability to reduce the soluble toxic form of chromium (Cr(VI)) to the less toxic and insoluble form (Cr (III)) [71,72,73]. This transformation is mediated by a reductase, ChrR, or when Cr(VI) is used as an electron acceptor [74,75]. Once Cr (VI) is reduced to Cr (III), it can be exported by the transmembrane efflux pump ChrA [76]. ChrR is a member of the NAD(P)H dependent FMN-reductase family that has a wide substrate specificity [74]. ChrR appears to be widely distributed among archaea (Figure 1). Interestingly, only a few archaeal species contain *chrA* (efflux transporter), suggesting that archaea may use different strategies to detoxify this metal, such as by activation of oxidative stress mechanisms, or via novel efflux pumps. Studies on the determinants controlling the activity of ChrR show reduction of uranium species could be accomplished by this protein [77]. The potential benefit of a single pathway that can reduce the action of multiple toxic metals could facilitate bioremediation efforts of heavy metal contaminated soils.

Uranium is a radioactive metal of growing interest as a carbon net-negative energy source, for defense-related weapons, and for general ecological considerations. However, in archaeal genomes there are as yet no uranium specific reductases or efflux transporters that can be identified. A recent study describes the formation of insoluble uranium precpitates using cultures of the hyperthermophilic crenarchaeote, *Pyrobaculum islandicus *[78] perhaps reminiscent of the well characterized reduction of this metal by bacterial taxa belonging to *Geobacter* and *Shewanella spp. *[79,80,81,82,83]. 

## 5. Environmental Applications Using Metal Resistant Archaea

There are increasing efforts underway to mitigate acid mine drainage (AMD) from active and abandoned mines. Similarly, there are ever increasing demands for metal commodities to meet the needs of growing societies. For this reason the relationship between metals and archaeal taxa is of great interest. A number of archaeal taxa have been isolated from such sites and their analysis has fostered a better understanding of the microbial community dynamics that occur in such environments using culture independent methods [84]. 

The role of thermoacidophilic archaea in biomining (bioleaching) of sulfidic metals is another area of growing interest [85]. Hyperthermophilic archaea have the capacity to immobilize metals, such as uranium [78] suggesting uses for bioremedation of contaminated sites. In addition to these benefits, genetic engineering tools for some archaea are established [86] and may lead to biological systems with metal-leaching specificity and with increased rates of metal solubilization. 

## 6. Concluding Remarks

Archaea are globally distributed microorganisms inhabiting extreme environments as well as environments rich in heavy metals. These features foster interest in their genomics. The abundance of archaeal taxa in established environments rich in heavy metals has important ecologic and environmental implications. Elevated temperatures arising from mining in the deep subsurface promise to enhance availability of new thermophilic and hyperthermophilic species with novel metal metabolisms. There are several outstanding questions that remain to be answered in this field. What is the overlap between the unique cell biology of archaea and metal resistance? What is the ancient and modern day role of archaea in biogeochemical process? What unexplored biodiversity remains to be discovered in these extreme environments. Future studies integrating the use of genetic systems with model organisms will be critical to establish cause and effect relationships about metal biology and the archaea. 
